# Physiological Responses and User Feedback on a Gameful Breathing Training App: Within-Subject Experiment

**DOI:** 10.2196/22802

**Published:** 2021-02-08

**Authors:** Yanick Xavier Lukic, Chen-Hsuan (Iris) Shih, Alvaro Hernandez Reguera, Amanda Cotti, Elgar Fleisch, Tobias Kowatsch

**Affiliations:** 1 Centre for Digital Health Interventions Department of Management, Technology, and Economics ETH Zurich Zurich Switzerland; 2 Universidad de Sevilla Sevilla Spain; 3 Centre for Digital Health Interventions Institute of Technology Management University of St.Gallen St.Gallen Switzerland

**Keywords:** breathing training, serious game, biofeedback, mobile health, mHealth, mobile phone

## Abstract

**Background:**

Slow-paced breathing training (6 breaths per minute [BPM]) improves physiological and psychological well-being by inducing relaxation characterized by increased heart rate variability (HRV). However, classic breathing training has a limited target group, and retention rates are very low. Although a gameful approach may help overcome these challenges, it is crucial to enable breathing training in a scalable context (eg, smartphone only) and ensure that they remain effective. However, despite the health benefits, no validated mobile gameful breathing training featuring a biofeedback component based on breathing seems to exist.

**Objective:**

This study aims to describe the design choices and their implementation in a concrete mobile gameful breathing training app. Furthermore, it aims to deliver an initial validation of the efficacy of the resulting app.

**Methods:**

Previous work was used to derive informed design choices, which, in turn, were applied to build the gameful breathing training app Breeze. In a pretest (n=3), design weaknesses in Breeze were identified, and Breeze was adjusted accordingly. The app was then evaluated in a pilot study (n=16). To ascertain that the effectiveness was maintained, recordings of breathing rates and HRV-derived measures (eg, root mean square of the successive differences [RMSSDs]) were collected. We compared 3 stages: baseline, standard breathing training deployed on a smartphone, and Breeze.

**Results:**

Overall, 5 design choices were made: use of cool colors, natural settings, tightly incorporated game elements, game mechanics reflecting physiological measures, and a light narrative and progression model. Breeze was effective, as it resulted in a slow-paced breathing rate of 6 BPM, which, in turn, resulted in significantly increased HRV measures compared with baseline (*P*<.001 for RMSSD). In general, the app was perceived positively by the participants. However, some criticized the somewhat weaker clarity of the breathing instructions when compared with a standard breathing training app.

**Conclusions:**

The implemented breathing training app Breeze maintained its efficacy despite the use of game elements. Moreover, the app was positively perceived by participants although there was room for improvement.

## Introduction

### Background

Slow-paced breathing has been shown to promote psychological well-being [1-3] and physiological outcomes [4-6]. It increases heart rate variability (HRV) [7], which is positively associated with levels of relaxation and cardiovascular health and can be beneficial for health outcomes such as stress [2,8], depression [3,9], anxiety disorders [10], hypertension [5,11], type 2 diabetes mellitus [12], and chronic pain [6]. Furthermore, slow-paced breathing training also shows promise for helping to control respiratory diseases such as chronic obstructive pulmonary disease [13]. As a result, the effectiveness of breathing training is often measured based on increases in HRV, which reflect activation of the parasympathetic nervous system and, thus, the level of relaxation [14].

These positive effects gave rise to many breathing training applications [15-18], digital experiences, and games that use breathing as a marker to help individuals reach a relaxed and mindful state [18,19] or manage specific health issues [20]. The core of these applications usually consists of animation providing periodic guidance based on an underlying breathing pattern that results in approximately 6 breaths per minute (BPM), for example, 4 seconds of inhalation, 2 seconds of exhalation, and 4 seconds of pause [4]. A small number of applications also use biofeedback, which promotes the effectiveness of breathing training [21,22]. Biofeedback is a technique in which individuals learn to adjust their behaviors by controlling certain physiological activities (eg, breathing rate). However, apps with biofeedback [16,20] often have limited scalability, as the acquisition of physiological data requires special hardware (eg, respiratory belt) to provide individuals with understandable real-time information to which they can respond. Moreover, the reach of many applications is even more limited, as their breathing training is branded as a meditative technique, which is rarely used by male and lower educated and physically inactive individuals [23]. Finally, it has been shown that although breathing training apps yield high numbers of installations, the retention rates and, thus, long-term adherence are very low [24].

To overcome the challenges of limited reach and long-term engagement, researchers and developers often draw knowledge from the game design literature to increase the experiential value in nongame domains, such as education [25], health [26], and information systems research [27,28]. Recently, researchers have started to develop design principles that rely on breathing information as sensory input for games [29]. Furthermore, applications have begun to provide interactive breathing training based on breathing information [16,20,30] or HRV measurements [18]. Although the latter provides information on the actual effect of the training, it is limited in providing responsive feedback. The reasons for this are that HRV is not a consciously controlled input, and because of its deferred nature, it cannot achieve a reaction in a fraction of a second. However, a real-time feedback loop in gameful breathing training requires such short reaction times. Although there are applications based on breathing-based biofeedback, they do not address the limitations on reach and scalability. The reason for this is that their designs focus on virtual reality [16] or desktop computer [20,30] setups that are not easily accessible to an individual.

Although the majority of the population owns a smartphone, the design of interactive mobile breathing training games remains sparsely explored. Furthermore, existing designs have their primary focus on stationary virtual scenes [16,20] instead of dynamic journeys that change with the training. Such constant visualization changes would be less repetitive when the breathing training lasts several minutes and when an individual performs it regularly.

There are 2 essential aspects to be considered when designing a biofeedback-based breathing training app: *biosignal* (ie, breathing patterns) detection and *biofeedback* visualization that is directly perceived by individuals. Designing such a visualization that incentivizes long-term adherence and is still effective is challenging. It is essential to ensure that the gameful design does not interfere with the actual objectives. Liu et al [28] used the term *meaningful engagement* to describe the relationship between the experiential and instrumental (nongame goal of the task) values of a gamified task, and they stated that both values should always increase together. This means that the gameful design should not introduce elements that may weaken the instrumental aspect of the task even if they increase the experiential value. Therefore, it is crucial to make task-specific design choices to increase the experiential value while maintaining or increasing the actual outcome of the underlying task. In the context of breathing training, gameful elements (experiential values) should not impair the effectiveness (instrumental value) of the training. Such impairments in the effectiveness could, for example, occur through overexcitation of the user.

### Objectives

**Figure 1:**
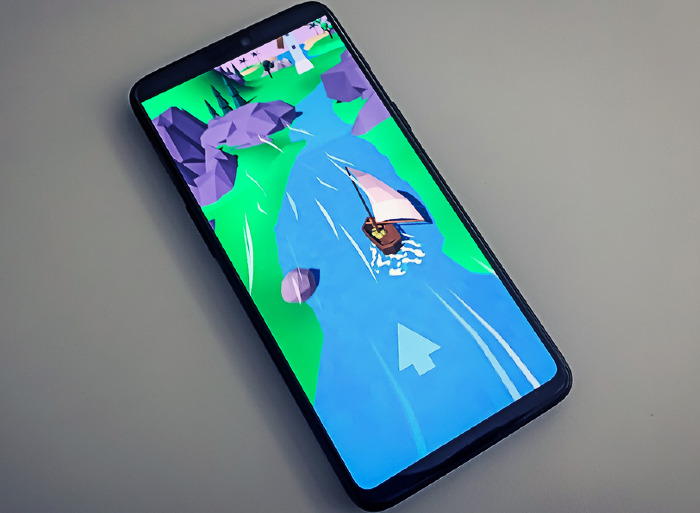
Our developed mobile gameful biofeedback breathing training Breeze running on a smartphone.

In a previous work, we introduced the gameful biofeedback breathing training *Breeze* [31] (Figure 1). In this study, we assessed the feasibility of detecting breathing patterns, that is, the *biosignal*, which is the first essential aspect of such breathing training. Although this study provided a high-level description of the mechanics of the visualizations and their acceptance in users, it did not provide detailed information about the design choices that guided the design of the second essential aspect of Breeze, namely, the *biofeedback* visualization. It also did not provide an in-depth analysis of its physiological efficacy. Consequently, this study aims to explain the design choices during the development process of Breeze and to provide a detailed analysis of physiological responses when compared with the standard breathing training. The long-term objective of such gameful breathing training is to foster long-term engagement. This study takes the first step by verifying that carefully designed gameful breathing training does not impair the physiological effects compared with the standard breathing training. It, therefore, asked the following research question: *Do gameful and nongameful breathing trainings trigger comparable physiological responses?* Given that gameful breathing training generated similar physiological responses, we asked the following research questions: *How are the different components of the developed gameful breathing training perceived, and what are the implications for its future development?*

Breathing training could be made gameful in various ways. We designed Breeze based on previous work on breathing training and biofeedback games and general design approaches used to induce relaxation. Physiological responses in participants of a pilot study were analyzed to answer the first research question. To answer the second set of research questions, we collected participant feedback regarding their perception of Breeze. The objectives of this study were as follows:

1. To present the development process of gameful and nongameful breathing trainings that share the same breathing pattern.
2. To assess the physiological responses to the gameful and nongameful breathing trainings.
3. To collect participant feedback on the gameful breathing training and to identify implications for future work.

## Methods

### Literature-Derived Design Choices

The main aim of the visual and interaction design is to foster relaxation and calmness in individuals while they remain motivated to continue. We, therefore, initially discussed fundamental building blocks for visual and interaction design (eg, colors, objects, game mechanics) and the psychological effects they trigger in individuals. These building blocks were then used together with existing design approaches for breathing training to produce a set of design choices for the development of our gameful breathing training.

#### Psychological Effects of Colors

Mehta and Zhu [32] found that blue colors triggered an approach and red colors triggered avoidance motivation. The use of blue colors, therefore, promotes more explorative and risky behavior, whereas the use of red colors results in a more vigilant and risk-averse state [33-35]. Whether this can be generalized to warm (eg, red, yellow) and cool (eg, blue, green) colors remains unclear. Nevertheless, additional research has indicated that cool colors have a calming and relaxing effect [36,37]. Thus, the use of cool colors, especially blue, was favored to induce more relaxation and calm in individuals. Furthermore, games that target the *explorer* player type [38] could potentially benefit from the enhanced explorative motivation of the individuals.

- Design choice 1. Use cool colors mainly to promote approach motivation and achieve a calming effect.

#### Environmental Influence on Well-Being

Research suggests that exposure to natural environments fosters relaxation and mental well-being [39,40]. Multiple projects have leveraged these findings [19,41]. Patibanda et al [16] confirmed this in the context of breathing training. They found that simple visuals of natural objects, such as trees, leaves, and water, helped participants relax and remain calm while playing a virtual reality game.

Nevertheless, the underlying mechanisms that induce relaxation in individuals when experiencing nature are still unclear. One influencing factor appears to be noise exposure in natural environments when compared with urban environments [42]. For example, a study showed that exposing stressed individuals to natural sounds improved their ability to recover from a stressful situation [43]. However, because we rely on the microphone of the mobile device for breathing detection, we may not be able to play sounds. Moreover, other theories provide suggestions that do not rely on sound. One states that exposure to nature activates the parasympathetic nervous system and thus reduces stress [39]. According to this theory, this activation could originate in the evolution of humankind because grassy environments with trees are usually a better source of food and shelter. On the basis of this line of argument, research has also suggested that people have an intrinsic preference for natural landscapes, in particular natural greenspaces, compared with urban landscapes [40,44].

However, research has shown that actual natural environments are not required to cause these effects. Researchers have found that simply showing images and videos of natural scenes was sufficient to reduce stress in participants [45].

To conclude, researchers are still investigating the underlying mechanisms of action. Nevertheless, studies have shown promising effects on stress, relaxation, and calmness levels of individuals when exposed to nature. From this, we derived that using a peaceful natural environment is beneficial by inducing a calming effect in individuals.

- Design choice 2. Use a natural and peaceful environment to induce a calming effect and a sense of well-being.

#### Increased Experiential Value Through Game Elements

The main purpose of increasing experiential value is to broaden the target audience for breathing training and to improve long-term adherence by engaging and motivating individuals. Classic approaches include adding game elements such as leader boards, achievements, and point gathering to otherwise monotonous tasks [28,46]. However, it is crucial to ensure that the employed game elements do not interfere with the underlying task and, for example, decrease its instrumental value [28], be it the amount of text produced by a writer or the health benefits for a patient. However, experiential value is affected by not only the employed game elements themselves but also the feel of the game dynamics. When physiological sensors influence the game state, this is especially important. Nacke et al [47] recommend mapping physiological sensors that have a direct impact on a game to actions in the virtual world. Sicart [48] defines actions (methods invoked by agents that interact with the game state) to be game mechanics. On the basis of these 2 references, we concluded that mapping direct physiological inputs to game mechanics can help improve the feel of game dynamics.

- Design choice 3. Integrate breathing guidance and biofeedback into the context as much as possible but never at the cost of comprehensibility.
- Design choice 4. Have the input from physiological measures reflected as game mechanics.

An essential part of increasing an individual’s enjoyment and thereby the experiential value is to add a kind of progression model toward a goal to help keep the individual motivated to carry on. For a task such as breathing training, a progression model is especially relevant as the individual is required to stay focused for a specified period. Many gamification approaches provide such a mechanism. A simple example would be to show a score at the end of a session. Although this can increase the willingness of an individual to return and try to reach a higher score [49], it does not necessarily motivate individuals during a session. It is, therefore, essential to give individuals a continuous feeling of progress throughout a session. One possibility would be to continuously change the appearance of an object or shift the viewport into new environments. In *Life Tree*, the saturation level of a tree provides a sense of progress [16]. However, one game session lasts approximately 2.5 min. It is questionable whether this progression model yields sufficient diversity with a longer playing time.

Games often achieve this by applying flow theory [50]. With regard to gameplay, the theory states that a game’s difficulty should gradually scale with the abilities of the individual playing it. This way, the individual is still challenged but not overwhelmed and thus attains flow in the activity. Although most research has focused on gameplay to drive flow, narrative engagement can achieve the same result. As previous research has shown, the concepts of flow, narrative engagement, and enjoyment are highly correlated and often inseparable [51]. The challenge in the context of gameful breathing training is to not overexcite the individual. As a result, changing difficulty levels and challenges can be difficult. Another solution could be to use embedded narratives to give the virtual world some more variety [52] and keep the individual engaged. These narratives do not have to be explicitly told (eg, through dialogues between characters); however, they can be hinted at and then left entirely to the imagination of the individual. A way of achieving this can be to design the virtual world in a way that it tells its story by itself, for example, by adding specific objects to it. We concluded that a continuous progression model is required that motivates individuals while not overexciting them. A possible solution could be to employ light forms of embedded narratives.

- Design choice 5. Provide a continuous feeling of progress toward a goal without overexciting the individual.

### System Design and Development

Our gameful breathing training Breeze uses a breathing training duration of 6 min and a 4-2-4-second inhale-exhale-pause breathing pattern, as in Russell et al [4]. The virtual world is set in a natural environment, where the individual helps a boat travel downriver by controlling the wind’s strength through breathing.

#### Implementation of the Design Choices

In the following paragraphs, we discuss how the design choices have been implemented in Breeze.

##### Design Choice 1 and Design Choice 2: Peaceful Natural Environment

The first 2 design choices, using natural environments and cool colors, are best addressed together. We made use of many natural objects (eg, trees, bushes, mountains) with mostly cool colors. For a simplistic look and to ensure that the app ran smoothly on mobile phones, we used meshes with a low polygon count and mostly flat shading.

##### Design Choice 3 and Design Choice 4: Integration of Guidance and Biofeedback

Wind is the main component that indicates the breathing pattern. We used tailed particles to visualize the wind. When the wind particles move in the opposite direction to the boat’s movement and toward the viewport, they indicate the inhalation phase. The exhalation phase is indicated when the wind particles move in the same direction as the boat and away from the viewport. During the pause phase, the wind has no particular direction and the number of particles is drastically reduced. To further help individuals stick to the breathing pattern, we added a semitransparent user interface (UI) element to the game, which also shows the current breathing phase (*arrow down*=*inhalation*; *arrow up*=*exhalation*; *horizontal line*=*pause*).

The wind’s strength reflects the breathing input. There is a set of mechanisms that determine how the breathing input affects the wind’s strength and, consequently, the biofeedback. The reaction of the game state is mainly dependent on the current breathing phase based on the periodic breathing pattern. If the system detects an inhalation or exhalation during the same breathing phase indicated by the breathing pattern, the game state is affected. In the following paragraph, we call these 2 cases *correct inhalation* and *correct exhalation*. It is important to note that we differentiate between whether a correct inhalation or exhalation occurs for the entire period of the corresponding phase indicated by the breathing pattern or just for a fraction of it.

Correct inhalation has 2 consequences. First, the wind’s strength increases. Second, the possible maximum acceleration for the next exhalation phase continuously increases until it reaches a predefined maximum value or the correct inhalation ends. During a correct exhalation, the wind’s strength also increases, and thus, the boat accelerates. The preceding inhalation phase thereby determines the acceleration strength. If correct exhalation does not follow correct inhalation, the boat still has additional acceleration by some small but perceivable amount.

To provide comprehensive visual feedback that is well incorporated into the virtual world, the wind’s strength influences a variety of other elements. The wind inflates and deflates the sail more or less depending on the wind’s strength. The amount of inflation then influences the boat’s speed, which is additionally accentuated by the size of the waves behind the boat.

The wind’s strength is portrayed by increasing the number of particles and increasing their speed.

##### Design Choice 5: Movement and Environment Design

**Figure 2:**
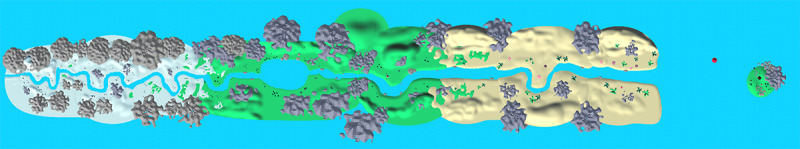
The environment design of Breeze is depicted. It starts in a snowy environment, passes into a grass-overgrown environment, and passes the sandy beaches before leading into the open sea.

The boat continuously travels through a changing environment to convey a feeling of progress. To prevent overexcitement that could impair the individual’s relaxation, we used a mostly static environment. However, the environment changes several times throughout 1 training session, thereby introducing some sense of narrative. The complete environmental design is depicted in Figure 2. The boat starts in a snowy landscape and advances into a grass-overgrown environment where the river merges into a small lake. Later, the boat passes the sandy beaches before sailing into the open sea. The boat may not reach the beaches or the open sea because the distance covered depends on the individual’s breathing performance. In the end, Breeze shows the distance achieved to give a sense of achievement. In the future, this score could also be used to motivate individuals to make improvements or could be compared with other people’s scores.

##### Pretest and Consequent Design Changes

**Figure 3:**
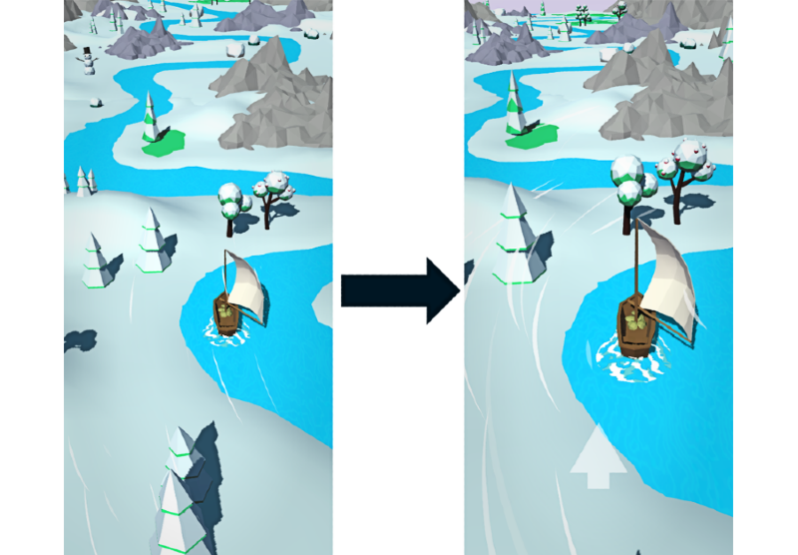
Breeze before (left) and after (right) applying design changes to address problems identified in the pretest. Specifically, the camera was moved closer to the boat, the wind particles were made more pronounced, and an additional user interface element was introduced to indicate the current breathing phase. There were also changes made to the boat’s acceleration behavior that are not specifically visualized.

The first version of Breeze was evaluated in a pretest. The findings led to 2 additional design choices and consequent adaptations to Breeze. The versions of Breeze before and after the pretest are depicted in Figure 3. In the pretest, 3 male participants, with an average age of 31 years (SD 2.45) and no previous knowledge about the app, were asked to conduct a breathing training session using Breeze and provide feedback. Even though they liked the overall setting and design of the experience (eg, 1 participant stated, “Nice graphics and landscape, the wind animation is good.”), they pointed out that it was not always easy to follow the breathing pattern. A participant stated that it needs “clearer guidance of breath in and out,” and another participant mentioned that the “animations are too small - difficult to follow.” Thus, the feedback mostly regarded the guidance component of Breeze. We attribute this to the fact that the guidance in this complex scene was not as pronounced compared with classic breathing training visualizations. Therefore, it was crucial to have guidance elements that were well separated from the background and in a concentrated area on the screen to form a clear focal point. Elements that cover a large surface are possible; however, they need to overlap this focal point. As a result, we made several adaptations to the visualization of the guidance system in Breeze:

1. The in-game camera was moved closer to the boat, which made the effects on the sail and the waves easier to identify.
2. The number of wind particles and the thickness of their trajectories were increased for clearer visuals.
3. A basic acceleration was introduced when the wind moved with the boat (exhalation) for better incorporation into the world’s context (following design choice 3).
4. An additional semitransparent UI element was added to indicate the current breathing phase (following design choice 3).

Because biofeedback and guidance are closely coupled, changes to the guidance system also require changes to the biofeedback mechanisms:

1. Because there is a basic acceleration of exhalation, the acceleration triggered by a correct exhalation was increased, so it remained noticeable (following design choice 3).
2. To fine-tune the feedback with regard to the basic acceleration, the maximum reachable acceleration was increased (following design choice 3).

#### Gameplay Overview

**Figure 4:**
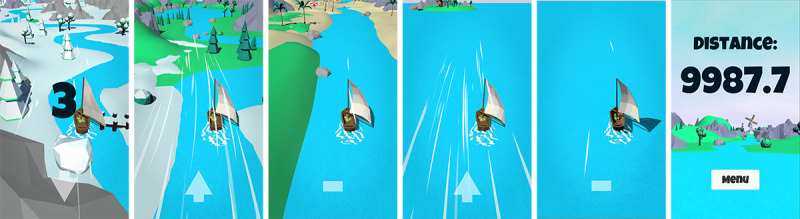
All phases of a breathing training session from Breeze are depicted from left to right: countdown, traveling from a snowy to grassy landscape, traveling from a grassy landscape to sandy beaches, reaching the open sea by passing a small island, traveling further into the sea, and finally, the score screen showing the distance traveled.

The game starts on a menu screen, where a breathing training session can be started. After clicking *start*, the view changes into game mode. The different stages of gameplay and the boat’s journey are depicted in Figure 4. In the beginning, the boat is standing still at a pier. At the end of a countdown, the 4-2-4 breathing pattern starts with an inhalation phase, and the boat starts moving at a constant pace. The individuals then have to adjust their breathing to the breathing pattern by following the guidance system. Depending on the ability of the individual to do so, the boat accelerates during exhalation. During the inhalation and pause phases, the boat’s speed always slowly decreases. The boat travels through the changing environment until 6 min have elapsed. When finished, the distance traveled is shown. A video of Breeze is provided in Multimedia Appendix 1.

#### Detection

The app uses a microphone to detect the different breathing phases (inhalation, exhalation, and pause). The algorithm is described in Shih et al [31].

#### Implementation

The gameful UI with biofeedback was created using the Unity game engine (version 2019.1.8). All models were designed and animated using 3D modeling software Blender.

### Pilot Study Design

**Figure 5:**
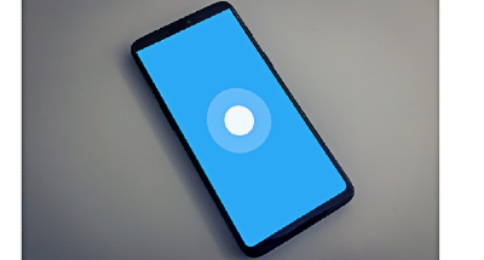
Visualization of the standard breathing training Circle. It served as a comparison for the impact of Breeze on participants' HRV measures. This way, the authors were able to ensure that Breeze yields similar physiological results although it employs game elements.

A pilot study was conducted to assess how Breeze was perceived (qualitative questions) and whether it reached a physiological response comparable with that of the standard breathing training (physiological measurements). The study was reviewed and approved by the ethics committee of ETH Zürich (ID: 2019-N-91). To this end, we compared Breeze with the standard slow-paced breathing training [4], which we refer to as *Circle* in this paper (Figure 5).

#### Physiological Measurements

The instrumental value of breathing training can be characterized as an increase in certain HRV measures. Our aim is to verify that Breeze has a comparable impact on HRV as that of Circle. We used *BioPac’s Smart Center Stand-alone BioNomadix Wireless System* to record heartbeat and derived several HRV measures from it. Furthermore, the breathing rate (BPM) and the abdominal respiration amplitude (ARA) were monitored through a respiratory belt. We used a sampling rate of 2000 samples per second for all measures. The different physiological measures that were studied in this study included the following [4,14,53]:

- Heart rate (HR): It is the number of heart beats per minute.
- High-frequency HRV (HF-HRV): It is the portion of the beat-to-beat intervals from 0.15 to 0.4 Hz. It is an indicator of the parasympathetic tone for breathing rates between 9 and 24 BPM [4].
- Low-frequency HRV (LF-HRV): It is the portion of the beat-to-beat intervals from 0.04 to 0.15 Hz. It is an indicator of oscillations in the baroreflex.
- Very-low-frequency HRV (VLF-HRV): the portion of the beat-to-beat intervals from 0.0033 to 0.04 Hz. This measure normally yields only small variations that are induced by breathing.
- Low- to high-frequency ratio (LF/HF ratio): It is the ratio between the low- and high-frequency portions of the beat-to-beat interval. This measure is thought to represent the sympathetic-vagal balance. However, this interpretation has been highly disputed [53]. Nevertheless, we include the measure in the analysis for completeness.
- Interbeat interval (IBI): It is the mean of the distances between different beats.
- Standard deviation of normal R-R intervals (SDNN): It is a representative measure of variability and is thus linked to the overall HRV.
- Root mean square of the successive differences (RMSSDs) between adjacent heartbeats: It is an appropriate indicator of parasympathetic tone when the breathing rate differs from 9 to 24 BPM. Therefore, RMSSD is an ideal measure for parasympathetic tone when at least 5 min of HR data are available.
- pNN50: It is the percentage of R-R intervals that are greater than 50 milliseconds away from adjacent intervals.
- Breathing rate (in BPM): It is the number of full respiration cycles per minute (inhalation, exhalation, and pause).
- ARA: It is the abdominal movement measured in volts.

Due to concerns regarding sphericity and the non-normal distribution of the baseline measurements as a result of the sample size, we used the nonparametric Friedman test as the omnibus test. Measures that yielded significant differences were then analyzed using the pairwise Wilcoxon signed-rank test. Compared with a previous work [31], we provide detailed physiological analyses in this study using all collected measures, consistent with established approaches [4].

We treated RMSSD as the main outcome for efficacy in this study. The reason for using RMSSD is its high correlation with other important measures, such as HF-HRV. Furthermore, in contrast to HF-HRV, RMSSD has the characteristic that it remains an appropriate indicator of parasympathetic tone when the breathing rate is lower than 9 BPM [4]. Nevertheless, as all the measures offer different perspectives on the outcomes, we provide a complete analysis for all of them. This allows us to discuss Breeze’s potential strengths and weaknesses in more detail. Consequently, the following hypotheses were tested:

1. There will be a difference in physiological response between Circle and the baseline assessment.
2. There will be a difference in physiological response between Breeze and the baseline assessment.
3. There will be no difference in physiological response between Circle and Breeze.

#### Self-Report Instrument

To gather feedback on Breeze, we asked participants 2 open questions at the end of the study:

1. What are positive aspects about using the sailboat breathing training?
2. What suggestions do you have for the sailboat breathing training?

The responses were then systematically coded by employing deductive coding by 2 of the authors [54]. All the code used and their descriptions can be found in Multimedia Appendix 2. After this initial coding round, we computed the raw agreement and Cohen kappa. Both coders then discussed their mismatches and were given 1 of the following 3 actions for each mismatch: favoring one coding over the other, merging the 2 codes, or finding a new coding.

#### Recruitment

A total of 16 participants (7 females) between 21 and 32 years old (mean 24.6, SD 3.44) were recruited at the first and last authors’ institutions. Participation was voluntary and compensated in local currency worth US $20.

#### Procedure

**Figure 6:**
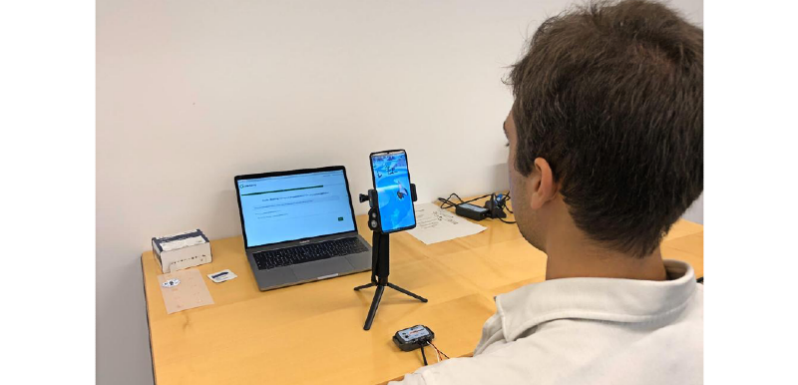
Experimental setup.

First, participants were welcomed in a quiet environment and asked to sit quietly for 6 min to collect data on their physiological baseline. Next, we sequentially presented Breeze and Circle to the participants. The participants used the 2 training sessions in random order. There was a washout period of 5 min between the training sessions to avoid carryover effects. For each training, the participants first familiarized themselves with the breathing training and then performed it for 6 min. After completing both trainings, participants filled in a qualitative questionnaire regarding their experience with Breeze. The study lasted approximately 50 min per participant. The study setup is shown in Figure 6.

## Results

### Physiological Responses to Breathing Trainings

**Figure 7:**
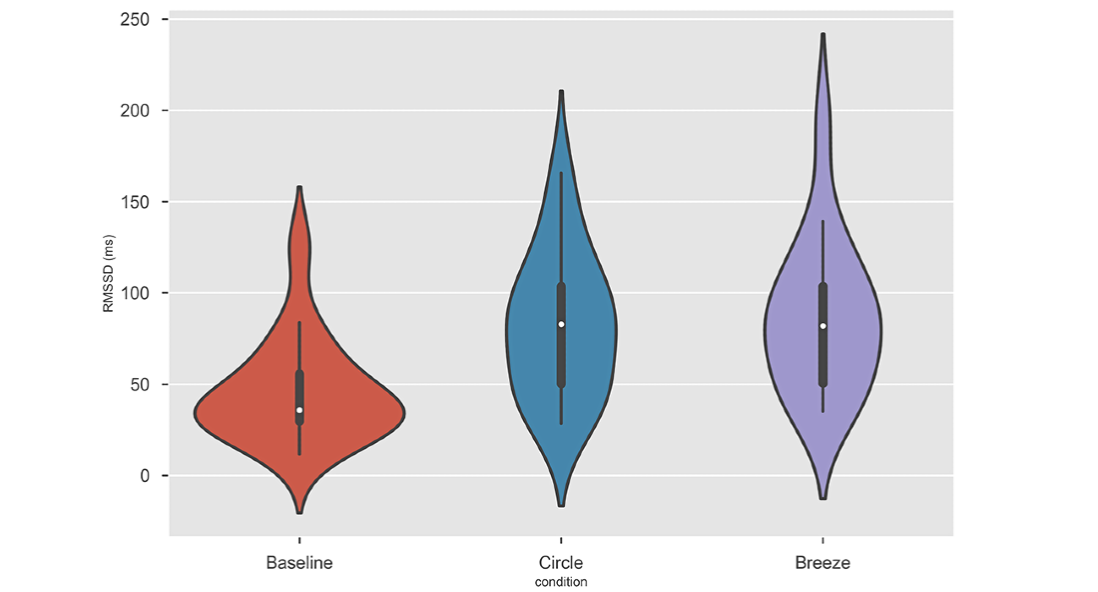
Violin plots depicting the distribution of the root mean square of the successive differences (RMSSD) measurements in all the 3 conditions. Each violin plot’s outer shape represents the complete distribution of the sample. In addition, standard boxplots with the medians represented by white points are included.

Because the distributions of some measurements did not pass checks for normality, nonparametric methods were used to analyze data. For the main outcome RMSSD, the measurement distributions in all conditions are shown in Figure 7. The mean, SD, median, and interquartile ranges for all measures in each condition (the baseline, Circle, and Breeze) are provided in Table 1.

Friedman tests were applied as omnibus tests to all measures across the 3 conditions. The results of the omnibus tests are shown in Table 2. At a significance level of α=.05, there were significant differences for all measures except VLF-HRV and ARA. All other measures were further analyzed using pairwise Wilcoxon signed-rank tests. The results are shown in Table 3.

**Table 1:** Assessment of physiological measures.

Variable	Baseline		Circle		Breeze	
	Mean (SD)	Median (IQR)	Mean (SD)	Median (IQR)	Mean (SD)	Median (IQR)
IBI^a^	837.25 (127.14)	836.00 (739.00-953.75)	857.00 (121.32)	842.50 (772.00-950.75)	843.75 (117.23)	835.50 (769.25-929.00)
HR^b^	73.31 (11.77)	72.00 (62.75-81.25)	71.50 (10.53)	71.50 (63.25-77.75)	72.31 (10.42)	71.50 (64.25-77.75)
SDNN^c^	53.65 (30.00)	45.30 (39.32-62.83)	102.52 (36.16)	106.60 (74.47-118.68)	111.81 (35.71)	104.65 (81.48-127.15)
RMSSD^d^	45.97 (28.05)	35.75 (29.65-55.62)	81.86 (39.20)	82.85 (50.17-103.55)	86.42 (41.56)	82.00 (50.53-103.50)
pNN50^e^	24.17 (20.86)	15.22 (8.56-37.66)	33.17 (15.42)	31.30 (27.41-39.15)	38.17 (16.16)	34.94 (27.84-53.48)
HF-HRV^f^	1073.00 (999.32)	812.00 (435.25-1269.50)	2067.06 (1826.66)	1762.50 (480.50-2861.75)	2696.62 (2830.56)	2186.00 (720.00-2795.75)
LF-HRV^g^	2530.00 (5189.85)	977.50 (563.75-1825.00)	8406.44 (5593.35)	7500.50 (4359.00-10,320.25)	10346.31 (6103.83)	7804.00 (6272.50-13,844.00)
VLF-HRV^h^	82.81 (140.09)	43.50 (18.25-72.50)	84.00 (127.36)	49.00 (20.00-70.75)	120.94 (119.60)	64.50 (37.75-182.50)
LF/HF ratio^i^	2.76 (4.83)	1.35 (0.82-1.64)	6.49 (4.65)	4.29 (3.11-8.36)	6.33 (3.99)	5.72 (2.91-9.09)
BPM^j^	12.23 (4.10)	11.83 (10.29-13.38)	5.99 (0.04)	6.00 (6.00-6.00)	5.96 (0.10)	6.00 (5.83-6.00)
ARA^k^	5.34 (2.40)	5.78 (3.33-6.58)	4.44 (3.09)	6.09 (1.34-6.44)	4.32 (2.84)	4.75 (2.08-6.62)

^a^IBI: interbeat interval.

^b^HR: heart rate.

^c^SDNN: SD of normal R-R intervals.

^d^RMSSD: root mean square of the successive differences.

^e^pNN50: percentage of normal R-R intervals greater than 50 milliseconds than their adjacent one.

^f^HF-HRV: high-frequency HR variability.

^g^LF-HRV: low-frequency HRV.

^h^VLF-HRV: very-low-frequency HRV.

^i^LF/HF ratio: low- to high-frequency ratio of heart rate variability.

^j^BPM: breaths per minute.

^k^ARA: abdominal respiration amplitude.

**Table 2:** Omnibus tests (Friedman) of within-subject effects.

Variable	Q (*df*)	*P* value
IBI^a^	7.625 (2)	.02
HR^b^	7.429 (2)	.02
SDNN^c^	25.125 (2)	<.001
RMSSD^d^	24.0 (2)	<.001
pNN50^e^	10.889 (2)	.004
HF-HRV^f^	15.5 (2)	<.001
LF-HRV^g^	19.5 (2)	<.001
VLF-HRV^h^	2.0 (2)	.37
LF/HF ratio^i^	14.0 (2)	<.001
BPM^j^	21.709 (2)	<.001
ARA^k^	1.175 (2)	.56

^a^IBI: interbeat interval (milliseconds).

^b^HR: heart rate (beats per minute).

^c^SDNN: standard deviation of normal R-R intervals (milliseconds).

^d^RMSSD: root mean square of successive differences between adjacent heartbeats.

^e^pNN50: percentage of normal R-R intervals that are greater than 50 milliseconds away from their adjacent intervals.

^f^HF-HRV: high-frequency heart rate variability (0.15-0.4 Hz).

^g^LF-HRV: low-frequency heart rate variability (0.04-0.15 Hz).

^h^VLF-HRV: very-low-frequency heart rate variability (0.0033-0.04 Hz).

^i^LF/HF ratio: low- to high-frequency ratio of heart rate variability.

^j^BPM: respiration cycles per minute.

^k^ARA: abdominal respiration amplitude (volts).

**Table 3:** Pairwise comparisons between baseline, Circle, and Breeze using the Wilcoxon signed-rank test.

Variable and condition			Comparison condition		Median of differences		Difference IQR		W statistic		*P* value^a^		RBC^b^
**IBI^c^**													
	Circle	Baseline		22.00		6.75 to 39.50		19		.01^d^		0.721	
	Breeze	Baseline		5.50		–8.25 to 23.25		52		.42^d^		0.235	
	Breeze	Circle		–18.00		–25.00 to –3.00		28		.04^d^		–0.588	
**HR^e^**													
	Circle	Baseline		–2.00		–4.00 to 0.00		15		.02^d^		–0.714	
	Breeze	Baseline		–0.50		–1.25 to 0.25		27		.36^d^		–0.308	
	Breeze	Circle		1.00		0.00 to 1.50		28		.12^d^		0.467	
**SDNN^f^**													
	Circle	Baseline		42.55		28.77 to 72.62		0		<.001		1.0	
	Breeze	Baseline		57.10		37.83 to 72.62		0		<.001		1.0	
	Breeze	Circle		9.45		–2.37 to 21.72		27		.03		0.603	
**RMSSD^g^**													
	Circle	Baseline		26.40		15.98 to 58.38		0		<.001		1.0	
	Breeze	Baseline		38.95		15.65 to 64.40		0		<.001		1.0	
	Breeze	Circle		1.15		–2.07 to 11.60		44		.23		0.353	
**pNN50^h^**													
	Circle	Baseline		13.28		2.55 to 16.45		24		.02		0.647	
	Breeze	Baseline		16.59		4.62 to 25.76		14		.003		0.794	
	Breeze	Circle		2.29		0.48 to 11.51		16		.01^b^		0.733	
**HF-HRV^i^**													
	Circle	Baseline		496.00		142.75 to 1758.25		12		.002		0.824	
	Breeze	Baseline		1095.50		403.75 to 2038.75		14		.003		0.794	
	Breeze	Circle		361.50		93.00 to 855.00		25		.02		0.632	
**LF-HRV^j^**													
	Circle	Baseline		5132.50		2173.25 to 8368.75		1		<.001		0.985	
	Breeze	Baseline		7027.50		4975.25 to 9084.75		1		<.001		0.985	
	Breeze	Circle		1665.00		–1134.75 to 3819.50		30		.05		0.559	
**LF/HF ratio^k^**													
	Circle	Baseline		2.76		1.65 to 6.67		16		.005		0.765	
	Breeze	Baseline		3.39		1.57 to 6.37		16		.005		0.765	
	Breeze	Circle		–0.31		–1.30 to 0.62		59		.670		–0.132	
**BPM^l^**													
	Circle	Baseline		–5.92		–7.38 to –4.29		1		<.001^d^		–0.985	
	Breeze	Baseline		–5.83		–7.38 to –4.29		1		<.001^d^		–0.985	
	Breeze	Circle		0.00		–0.17 to 0.00		8		.30^d^		–0.429	

^a^Reported *P* values are exact wherever possible.

^b^Effect size is reported through the rank biserial correlation.

^c^IBI: interbeat interval measured in milliseconds.

^d^*P* value was calculated through normal approximation because of ties.

^e^HR: heart rate measured in beats per minute.

^f^SDNN: standard deviation of normal R-R intervals measured in milliseconds.

^g^RMSSD: root mean square of successive differences between adjacent heartbeats.

^h^pNN50: percentage of normal R-R intervals that were greater than 50 milliseconds away from their adjacent intervals.

^i^HF-HRV: high-frequency heart rate variability (0.15-0.4 Hz).

^j^LF-HRV: low-frequency heart rate variability (0.04-0.15 Hz).

^k^LF/HF ratio: low- to high-frequency ratio of heart rate variability.

^l^BPM: respiration cycles per minute.

### Coded User Feedback

This section summarizes the information gathered through the coding of participant feedback. The codes used are *Gamification*, *Visualization*, *Guidance*, *Biofeedback*, *Circle versus Breeze*, *Relaxation* *Effect*, and *To add*.

The initial coding round, performed by 2 of the authors as independent coders, yielded a raw agreement p_o_ of 0.843 and Cohen κ of 0.812, indicating almost perfect agreement [55]. The main difference came from an inherent overlap of the code To add and the other codes. For example, coder 1 often only assigned the code Gamification when a participant criticized a game element and proposed changing it, whereas coder 2 favored the code To add in such circumstances. The coders came to a consensus by merging all but one mismatch, thus assigning both codes. In one case, they decided to use the coding provided by coder 1.

Because the narrative game element is mainly implemented through visualization, we combined the codes *Gamification* and *Visualization* in one section in the subsequent sections. The information yielded by the code *Circle versus Breeze* is incorporated into specific topics. Any quantitative numbers were calculated by counting the number of participants who made a statement that was assigned a specific code.

#### Gamification and Visualization

Thirteen participants wrote in their feedback that they enjoyed specific game elements or gamification overall. They mainly highlighted the score at the end and liked the playfulness compared with Circle. Two participants suggested increasing the gamification level by, for example, providing the score during breathing training. Another 4 expressed interest in receiving more breathing statistics at the end. Two participants explicitly mentioned that Breeze is more exciting than Circle: for example, “The feeling of accomplishment after doing the exercise in comparison with the feeling of nothing after the circle version.” Fourteen participants made supporting statements about the visualization and the narrative of the breathing training. These statements ranged from specifically visual standpoints, “The sailboat looks great,” to the appreciation of the narrative context, “The sailing boat topic for relaxation is awesome!” The natural setting of the virtual world was appreciated as calming and relaxing. Despite overall positive feedback regarding the visualization and narrative, 11 participants suggested improvements. One participant felt that the app was more appropriate for children than for adults, even though they liked the graphics overall. However, many improvement suggestions were given regarding the UI guidance symbols, such as “cooler symbols” or “It made the visuals redundantly cluttered.” Only one participant perceived that the gameful elements would corrupt their breathing, as they gave them the urge to test the limits of the app rather than follow the training correctly.

#### Guidance

Five participants positively emphasized the guidance in Breeze. One even felt that it was easier to follow than the standard breathing training: “easy to follow, easier than the circle.” Six participants had some ideas for improvement. One participant pointed out that the exhalation phase of the 4-2-4 breathing pattern is too short according to their personal preference. Three of them had difficulties following the guidance itself. The main issue was to anticipate the change between 2 breathing phases. One of them stated regarding the guidance elements: “they can’t be easily anticipated like the circle version.” Another participant who experienced similar issues suggested combining the circle animation with the sailboat to arrive at clearer guidance. Nevertheless, the majority of the participants were able to follow the training, even though it was surprising for some of them: “I thought it would be little distracting at first, but it was not. I was able to follow the exercise properly.”

#### Biofeedback

Because the biofeedback was triggered by the individuals’ input to the app, it was closely linked to feedback regarding overall gamification. Nevertheless, 5 participants explicitly pointed out that they liked receiving biofeedback. One participant even stated, “I feel the feedback system helps you be more relaxed.” This was in contrast to 2 other participants who felt that their breathing was not accurately detected.

Nevertheless, 3 participants suggested some improvements. One participant would have liked to have received statistics about their breathing performance during and after the breathing training. The other 2 suggested that the influence of the breathing input was more pronounced in the visualizations. One stated, “Improve the effects when the user is breathing, so they can better feel they are moving the boat.”

#### To Add

Five participants stated that they would have liked to have some background music and sound effects. One suggested using music as an additional measure to promote relaxation. Another participant thought that adding specific sound effects to the biofeedback could be beneficial: “Sound feedback to further motivate and reassuring the user that he's breathing correctly.” Another aspect mentioned by the participants was to add an element that lets the user know the remaining time for the current session. Furthermore, one participant suggested not only showing the score at the end but also setting a leaderboard to set a relative benchmark for their performance.

## Discussion

### Physiological Responses

The breathing rates of participants for the standard breathing training and Breeze clearly showed that both were effective in guiding slow-paced breathing with 6 BPM. The HRV measurements also showed an effect in both conditions. The differences between the conditions for these measurements were also consistent with previous work [4]. The only exception was that we found a significant difference in p50NN. Analysis of RMSSD showed that both conditions had a significant effect when compared with the baseline measurements. Comparing Breeze with the standard breathing training showed no significant difference. This conclusively confirmed the hypotheses for RMSSD. Furthermore, the comparison with the baseline yielded a perfect rank biserial correlation, supporting the claim of the presence of a large effect for both the standard breathing training and Breeze when compared with the baseline. The nonsignificant difference between Breeze and the standard breathing training with a very small median of differences was in line with the objective of reaching a comparable relaxation effect with Breeze.

The standard breathing training was able to slightly but significantly increase IBI and reduce HR, whereas Breeze did not yield a significant difference from the baseline for either measure. Although these measures were not the main outcome, they could have positively influenced HRV measures. However, important HRV measures (eg, HF-HRV, SDNN, or p50NN) yielded results comparable with those of RMSSD. Comparisons of the 2 conditions sometimes favored Breeze. Most notably, the HF-HRV measures were significantly higher for Breeze. Although the limited sample size does not allow for any conclusive reasoning, this should be further investigated with a larger sample.

Although VLF-HRV measures were elevated with both breathing trainings, they did not significantly differ from the baseline, consistent with previous work [4]. In addition, for ARA, no significant differences were found, which was also in line with previous work [4]. In our case, Breeze yielded slightly lower values than the baseline and the standard breathing training. This indicates that the participants did not breathe as much into the abdomen when using Breeze. Because this was driven by a small number of participants, we attribute this to the finding that Breeze’s breathing guidance requires further improvements. In addition, as stated by one participant, another factor could be that the game elements may have caused some participants to feel the urge to experiment with their way of breathing. Nevertheless, the overall findings support the claim that Breeze had comparable effects on HRV as those of the standard breathing training. From this, we conclude that Breeze achieved the main objectives of slow-paced breathing training despite the use of richer graphics and game elements.

### User Feedback

In this section, we discuss participants' feedback regarding Breeze. This is discussed in relation to the design choices that guided the implementation of Breeze.

#### Cool Colors and Natural Setting

The qualitative feedback shows that participants valued the environment and sometimes even explicitly pointed out the relaxing factor of nature. Thus, design choice 1 and design choice 2 appeared to be effective strategies to foster relaxation in individuals and were potentially beneficial for the instrumental value. They also contributed to an increase in the experiential value for several participants. These results support existing studies regarding color [36,37] and nature [39,40,44] perception and apps [16,19,41] already relying on the calming effect of nature.

#### Guidance and Biofeedback

The guidance and biofeedback that we implemented in Breeze were mainly driven by design choice 3. If not done well, it could have led to unclear guidance and biofeedback, thereby impairing the instrumental value or resulting in clear but incongruous guidance and biofeedback that breaks the immersion. Poor implementation could have even decreased the experiential value.

In Breeze, the increase in the wind’s strength and acceleration of the boat appeared to be adequate biofeedback for correct breathing. However, periodic guidance through the wind and UI symbols seemed to be the weakest component. Although they provided sufficient guidance to correctly follow the breathing training, several participants did not like the UI symbols, even though some others valued them as additional help. Nevertheless, the UI symbols violated design choice 3, as they were not neatly incorporated into the virtual world. The use of these UI symbols was, therefore, a design flaw within Breeze that triggered some participants’ responses. However, the participants who stated that they mostly relied on the UI symbols for guidance also often provided positive remarks about the movement and acceleration of the boat based on their breathing. Thus, they were still able to correctly follow breathing training through the symbols and consider biofeedback. Nonetheless, we plan to remove the UI symbols in favor of an additional guidance system that is well incorporated into the app’s world and, thus, improve compliance with design choice 3 (eg, more pronounced guiding animation by the sail).

User interactions with the experience were heavily influenced by design choice 4. As a result, this design choice is strongly linked to biofeedback and the gamification of breathing training. Being able to influence the experience through breathing at the right moment and consequently receiving biofeedback was valued by many participants. This supports the underlying findings of previous work that sensor inputs should be reflected as actions in the game [47]. The participants who expressed problems with biofeedback mostly stated that they did not feel that they had an impact on the app through their breathing. We do not attribute this to bad biofeedback design but to imperfect breathing detection for these individuals, which is possible based on some people’s breathing (eg, exceptionally silent inhalation or exhalation). However, this is not the focus of this paper and is discussed in detail in Shih et al [31], and it remains a major priority for our future work.

#### Progression Model

The implementation of design choice 5 was generally well received. Many participants explicitly mentioned that they liked the continuous progression through the virtual world. Several participants also had a sense of achievement when the distance traveled was presented, which supports the existing gamification literature regarding the motivational effect of extrinsic rewards [56]. Participants’ statements for potential improvements to the perceived progression were manifold, and various additional game elements should be considered [57]. A possibility would be to give the progression more meaning by providing game elements such as leaderboards or statistics in the end. Another possibility would be to provide more context to the progression by showing the remaining time in a well-integrated way or providing the live score to further motivate individuals. The progression model would also play a major role if the app is expanded with additional levels and other motivational aspects to further increase engagement. Such enhancements would then allow experiments with different approaches to achieve flow [50] in individuals and determine the boundaries before a level of overexcitement that impairs the instrumental value is reached.

#### Add Sound

Many participants stated that they wanted sound-based guidance and feedback. Using sound would be very challenging for the detection algorithm when the sounds are output through the smartphone's built-in speaker. However, this would be eliminated when using headphones. Therefore, the integration of sound-based guidance and feedback should be further investigated.

#### Overall Feedback

We also found that participants liked the gameful breathing training and that many participants stated by their own initiative that they preferred the gameful breathing training over the standard one. Furthermore, the participants liked that their breathing performance was reflected in the app. Combining these findings, we conclude that mobile gameful breathing training is a feasible endeavor. Nevertheless, there is still room for more game elements and improvements in Breeze, especially regarding guidance quality.

### Limitations

The ultimate goal of Breeze is to increase long-term adherence. However, the results of this study do not allow us to conclude whether its current state would improve the long-term adherence relative to the standard breathing training, as it has only been used in a single session.

This study also assumes that, based on previous work, richer visualizations and game elements increased the experiential value. Although qualitative feedback indicated that an increase in experiential value was achieved for many participants, additional studies are required to substantiate this claim in the context of breathing training.

In addition, some participants felt that biofeedback helped them relax. However, this would require an additional experiment in which the measurements of 2 training sessions would be compared—1 with and 1 without biofeedback. Furthermore, the participants did not hold the smartphone in their hands, which could affect how the app is perceived and the ability to relax. However, as this was the same for Breeze and the standard breathing training, this does not limit the comparison across conditions.

Moreover, such breathing training would require an additional tutorial or onboarding phase when deployed in a nonlaboratory setting. However, a majority of the participants did not have any problems understanding the functions of Breeze after receiving a short introduction.

Finally, the fact that Breeze relies only on a smartphone makes it highly scalable. However, it should be mentioned that hardware that is specifically designed for breathing monitoring may yield more information-rich and reliable data. For example, a respiratory belt also provides information on whether the abdomen is moving correctly while breathing. This would allow more detailed feedback, but at the expense of scalability.

### Future Work

With regard to the acceptance, effectiveness, and long-term adherence of breathing training such as Breeze, we plan to test it in different patient populations that require breathing training for different purposes, including, for example, students who are affected by mental health issues, want to reduce stress, or want to benefit from the general health benefits of breathing training. Other planned target populations are patients with cancer [58,59] and hypertension [60] who use breathing training as part of their complementary treatment.

We also plan to assess whether biofeedback has a beneficial effect on the efficacy of breathing training with Breeze. This can be achieved through an experiment in which breathing training is conducted with and without biofeedback.

It can be expected that in certain populations, designs other than the Breeze design would be more appealing. Thus, it is crucial to develop a design process that closely interacts with targeted populations [30]. Designing for specific target groups may lead to the necessity of partially or completely redesigning the breathing training app. In the process, we plan to consolidate and enhance the design choices of this study not only generally but also specifically to certain health outcomes. We plan to achieve this by leveraging feedback from individuals and using existing literature about designing technology to help with particular health outcomes (eg, for mental health and depression [61,62]).

### Conclusions

In this study, we explained 5 design choices that guided the development of our mobile gameful breathing training Breeze. It was developed through 2 iteration cycles featuring a pretest and an evaluation in a pilot study with 16 new participants. The results yielded overall positive qualitative feedback. In addition, physiological measurements showed that Breeze can guide participants to follow a predefined breathing pattern and, in the process, raise their HRV and, thus, trigger health benefits. The use of Breeze reached a physiological response in participants comparable with that observed in the standard breathing training. Breeze achieved this despite the use of richer graphics and game elements, which should ultimately lead to an increased experiential value and potentially improve engagement.

## Appendix

Multimedia Appendix 1 Breeze video.

Multimedia Appendix 2 Coding table.

## Abbreviations

ARA: abdominal respiration amplitude
BPM: breaths per minute
HF-HRV: high-frequency heart rate variability
HR: heart rate
HRV: heart rate variability
IBI: interbeat interval
RMSSD: root mean square of the successive difference
SDNN: standard deviation of normal R-R intervals
UI: user interface
VLF-HRV: very-low-frequency heart rate variability
