# Supplementary material for: Physiological Responses and User Feedback on a Gameful Breathing Training App: Within-Subject Experiment
Source: JMIR Serious Games. 2021 Feb 8;9(1):e22802. doi: 10.2196/22802 (PMC7899808; doi:10.2196/22802)
Supplement: Multimedia Appendix 2 [file games_v9i1e22802_app2.docx]

## Multimedia Appendix: Coding Table.

| **Code** | **Concept** | **Example** |
| --- | --- | --- |
| Gamification | Includes any comments and suggestions regarding any gamification element that are in Breeze or should be added in the opinion of the participant. | P16: “I liked the score to see how well I am breathing.”  P10: “I would like see the real time score each time I breathe.” |
| Visualization | Any comments concerning the visuals of Breeze. This includes the colors but also the 3D models, the level design, and the overall setting. | P6: “I liked the variety of the environment I was sailing into.” |
| Guidance | Comments about how the guidance is implemented, how well it was understood, and how easy it was to follow. It also includes the appropriateness of the chosen breathing pattern. | P1: “I thought it would be little distracting at first, but it was not. I was able to follow the exercise properly.”  P8: “The rhythm 4-2-4 seconds was not ideal for me, as I would naturally exhale for a longer time.” |
| Biofeedback | Comments about the biofeedback mechanisms. | P7: “I feel the feedback system helps you be more relaxed." |
| Circle versus Breeze | Comparisons between the circle condition with Breeze. | P12: “I liked that something was happening on the screen i.e. it was not as boring as the circle version.” |
| Relaxation Effect | Responses that state something about whether they felt relaxed because of Breeze, but also statements whether the design is adequate for a relaxation exercise. | P6: “The sailing boat topic for relaxation is awesome!” |
| To add | Suggestions to add new elements to Breeze. | P3: “Final score ranked among others (normal, excellent performance, low performance, mean references)”  P14: “Soundfeedback to further motivate and reassuring the user that he's breathing correctly.”  P10: “I would like to see the time remaining to complete the exercise” |
